# Visual Dysfunctions in Mild Traumatic Brain Injury: A Focus on Accommodative System Impairments

**DOI:** 10.3390/life15050744

**Published:** 2025-05-06

**Authors:** Nawaf M. Almutairi

**Affiliations:** Department of Optometry, College of Applied Medical Sciences, Qassim University, Buraydah 51452, Saudi Arabia; nm.almutari@qu.edu.sa

**Keywords:** mild traumatic brain injury, diffuse axonal injury, neurometabolic cascade, visual impairments, oculomotor abnormalities, accommodative dysfunction

## Abstract

**Background**: Mild traumatic brain injury (mTBI) is a prevalent neurological condition that results in various physical, emotional, and cognitive impairments. The most common are visual impairments, which affect vision’s perceptual, motor, and sensory aspects. **Objective**: This paper analyzes the pathophysiology of mild traumatic brain injury (mTBI) and its effects on visual and oculomotor functions, focusing on the deficits of the accommodative system and their underlying mechanism. **Findings**: mTBI frequently causes diffuse axonal injury, resulting in abnormalities of the neurometabolic cascade that impact the brain’s visual regions. Accommodative anomalies, including insufficiency, infacility, and spasm, are markedly more common in mTBI patients than in the general population. These deficiencies present as a notable delay in accommodation response, diminished peak velocity, and compromised dynamic responses, possibly due to sensory and motor disturbances. **Conclusions**: Accommodation disorder is a significant but under-examined component of visual sequelae related to mTBI. Future research should concentrate on the sensory and motor factors contributing to these deficiencies to enhance diagnostic precision and customize rehabilitative strategies.

## 1. Introduction

Mild traumatic brain injury (mTBI) is a growing public health concern, particularly among military personnel, athletes, and individuals involved in motor vehicle accidents or falls [1,2,3,4]. Although classified as “mild,” the neurological and functional consequences of mTBI can be significant and long-lasting [5]. Among the most common and persistent sequelae are visual disturbances, which are reported in up to 90% of individuals with mTBI. These visual sequelae can affect the oculomotor and non-oculomotor systems, severely impacting quality of life and day-to-day functioning.

One of the most understudied yet clinically relevant aspects of post-mTBI visual dysfunction is accommodative impairment. The accommodation system, which controls the eye’s ability to focus on near objects, depends on finely tuned sensory and motor mechanisms. Disruption of these mechanisms through diffuse axonal injury or neurometabolic disturbances can lead to symptoms such as visual fatigue, headaches, and reading difficulties—symptoms frequently reported by individuals with mTBI.

This review provides an in-depth synthesis of the current understanding of mTBI’s impact on the accommodative system. It explores the underlying pathophysiological mechanisms, the prevalence and clinical features of accommodative dysfunctions, and how theoretical accommodation models can help explain the observed clinical phenomena. By consolidating the literature and highlighting areas of research gaps, this review aims to support clinicians and researchers in improving diagnosis and tailoring rehabilitative strategies for patients with mTBI.

## 2. Methodology

This review was conducted using a narrative approach to synthesize current literature on accommodative dysfunction associated with mild traumatic brain injury. A comprehensive search of peer-reviewed articles was performed across several electronic databases, including PubMed, Scopus, Web of Science, and Google Scholar, covering publications from 2000 to 2025. The search terms included combinations of the following keywords: “*mild traumatic brain injury*”, “*accommodation*”, “*accommodative dysfunction*”, “*visual impairment*”, “*oculomotor disorders*”, and “*diffuse axonal injury*”.

Studies were included if they (1) focused on human subjects with clinically diagnosed mTBI, (2) examined accommodative or oculomotor function post-injury, and (3) were published in English. Both objective and subjective assessments of accommodation were considered. Exclusion criteria included studies with participants with moderate or severe TBI, pre-existing visual conditions unrelated to TBI, or insufficient methodological detail.

Priority was given to empirical studies, meta-analyses, and reviews that provided insights into the physiological, clinical, or theoretical understanding of accommodation in mTBI. Reference lists of selected articles were also hand-searched to identify additional relevant studies. The synthesis emphasizes recurring patterns, gaps in research, and implications for clinical practice.

## 3. Mild Traumatic Brain Injury

### 3.1. Definition

The definition of mTBI is based mainly on the criteria of the injury manifestations. The World Health Organization (WHO) task force recently used a common criterion to define mTBI as “an acute brain injury resulting from mechanical energy to the head from external physical forces. Operational criteria for clinical identification include (i) one or more of the following: confusion or disorientation, loss of consciousness for 30 min or less, post-traumatic amnesia for less than 24 h, and/or other transient neurological abnormalities such as focal signs, seizure, and intracranial lesion not requiring surgery; (ii) Glasgow Coma Scale score of 13–15 after 30 min post-injury or later upon presentation for healthcare. These manifestations of mTBI must not be due to drugs, alcohol, or medications, caused by other injuries or treatment for other injuries (e.g., systemic injuries, facial injuries, or intubation), caused by other problems (e.g., psychological trauma, language barrier, or coexisting medical conditions) or caused by penetrating craniocerebral injury” [6].

### 3.2. Mechanism and Neuropathology of mTBI

The primary neural-pathological feature in mTBI is diffuse axonal injury (DAI) [7]. There is considerable evidence showing that linear and rotational acceleration of the brain during the moment of impact is the primary cause of concussive injuries. Linear acceleration has been shown to correlate with the peak pressure within the brain, which may lead to brain damage. On the other hand, the rotational acceleration can lead to shear forces across the brain tissues [8].

**Figure 1:**
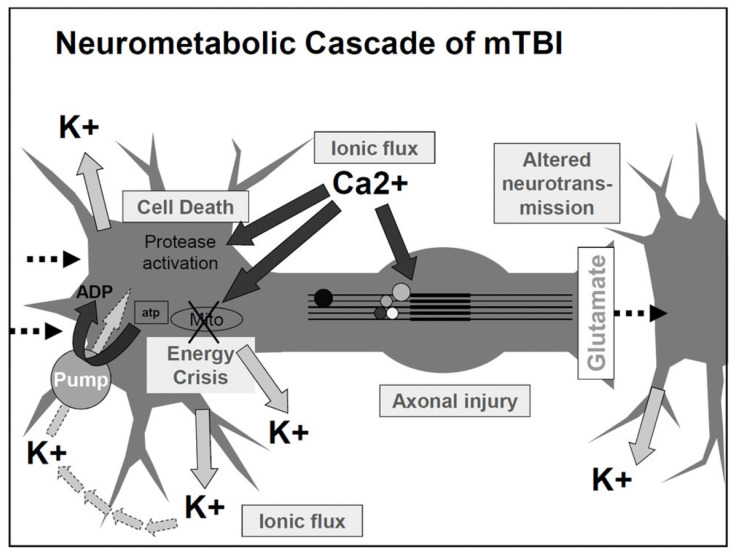
Diagram of the acute cellular biological processes occurring after concussion/mild TBI. Reproduced with permission from Giza, C.C., and Hovda, D.A., *The New Neurometabolic Cascade of Concussion*, Neurosurgery, Vol. 75, October 2014; published by Wolters Kluwer Health, Inc., 2014 [11].

Biomechanical injury to the brain leads to neural membrane deformation. This deformation could cause neurochemical and neurometabolic events, including ionic imbalance, such as excessive potassium efflux to the extracellular space, an influx of calcium ions, and glutamate release, resulting in massive cell depolarization. Subsequently, the brain attempts to restore the ionic balance by requiring high energy consumption, eventually reducing the stored energy level and causing more cellular energy crises [9]. In addition to the increase in glucose consumption, calcium accumulation in the intracellular space compromises the mitochondrial function, causing an impairment of oxidative metabolism. Eventually, this impairment of the mitochondria functions reduces energy (ATP) production [10]. Figure 1 shows the proposed neurometabolic cascade after concussion.

## 4. MTBI Impact on Oculomotor and Non-Oculomotor Function

### 4.1. MTBI General Sequalae

In the event of global injury caused by TBI, an isolated dysfunction is infrequent. Although no structural brain damage is detected on conventional imaging scans, mTBI affects a wide range of brain areas, causing a spectrum of emotional, cognitive, physical, and behavioral dysfunctions and could lead to many symptoms that affect the quality of life and well-being.

Cognitive dysfunction, which could persist for years after the original TBI event, is one of the most debilitating sequelae. The short-term cognitive problems may include amnesia, which could lead to disorientation, memory impairment, and confusion [12,13]. The long-term cognitive dysfunction may consist of impairments in attention, information processing, communication, visuospatial processing, and awareness of the deficits [14]. A recent review by McInnes and coauthors found that a large proportion of individuals with a single mTBI will continue to show measurable impairment in several cognitive areas, including executive function, learning/memory, attention, processing speed, and language function long after the initial injury [15]. Indeed, physiological correlation studies found that DAI is associated with underperformance in cognitive testing [16].

Other commonly reported post-injury symptoms include headache, sleep disturbance, and fatigue. Headaches are frequently described as migraine-like or tension-type in nature [17]. According to a recent systematic review by Cancelliere et al. (2023), fatigue and sleep disturbances are prevalent self-reported symptoms in adults with mTBI 3–6 months post-injury, often co-occurring with other physical and cognitive complaints. While these symptoms are commonly reported together, the mechanisms underlying their expression may differ. Fatigue may occur independently as a direct consequence of neural injury, or it may be exacerbated by sleep disruption, cognitive strain, or psychological factors. The review highlights that persistent fatigue and sleep disturbances contribute significantly to long-term disability, underscoring the need for comprehensive assessment and individualized management strategies in mTBI rehabilitation [18].

### 4.2. MTBI Impact on the Non-Oculomotor Visual System

The visual system occupies many areas of the brain. An injury to one or multiple brain areas can result in sensory, motor, perceptual, and refractive anomalies. Therefore, combining more than one of these problems can make testing and treatment more challenging. The common non-oculomotor visual problems reported in the literature are photosensitivity, spatial localization deficit, motion hypersensitivity, vestibular, and higher-order visual information processing problems [19,20].

Photosensitivity or photophobia is one of the most common visual symptoms caused by mTBI. Clinical reports estimate that 50–55% of patients with mTBI have photosensitivity [21,22]. Photosensitivity has gained significant awareness in the research field over recent years. Photosensitivity can be painful and limit the activity of daily living (ADL) [22]. Even normal light levels can trigger severe headaches and other somatosensory symptoms in patients who suffer from photosensitivity after mTBI. These photosensitivity symptoms can be exacerbated by certain light conditions such as fluorescent lighting or computer screen usage [21]. The exact mechanism of photosensitivity after mTBI has not been fully elucidated in the literature. Currently, there are three postulated pathways proposed to be involved in causing this response. The first pathway is the projection of retinal ganglion cells to the trigeminal (cranial nerve V) nucleus and then to the ophthalmic division (V1), which may be responsible for nociceptive afferents from the eye and orbit [23]. The second pathway involves the intrinsically photosensitive retinal ganglion cells (ipRGC) that project to part of the thalamus, which also receives intracranial input. The third pathway is a projection from ipRGC cells found in rodents’ iris that activate trigeminal ocular afferents [23].

Currently, there is no major randomized controlled trial for treating photosensitivity. However, the common clinical treatments include sunglasses and color-tinted spectacles. Sunglasses can reduce photosensitivity by diminishing overall light transmittance to the eye. At the same time, color-tinted glasses appear to alleviate photosensitivity through selected filtering of the wavelength spectrum and/or luminance reduction. Good et al. found that rose-tinted lenses reduce photosensitivity in children with migraine headaches [24]. These lenses filter the short-wavelength light in fluorescent lighting. Although effective for some, the response to color filters is not universal among all patients with mTBI. Even for those who showed treatment effects, mTBI patients vary in their specific color preference and appear to be more individualized. A recent study found that blue and green lenses reduced photosensitivity in 75% of a sample of 33 subjects with post-concussion syndrome [25]. Since the mechanism of photosensitivity is still unclear, no pharmacological intervention exists to treat photosensitivity.

### 4.3. MTBI Impact on the Oculomotor System

Oculomotor dysfunctions in mTBI include deficits in version, vergence, and accommodation systems. Clinical studies and reviews have reported different oculomotor disorders’ prevalence. Ciuffreda et al. reported in a retrospective study that 90% of TBI patients with vision-related symptoms were diagnosed with one or more oculomotor dysfunctions [26]. Oculomotor dysfunctions in TBI can be further divided into strabismic and non-strabismic. While causes of oculomotor dysfunction can be cranial nerve palsies in severe TBI, they are less likely to occur in mTBI [27].

Saccadic and pursuit eye movement dysfunctions are common after mTBI. In one retrospective study, Capo’-Aponte et al. reported that 30% and 60% of mTBI patients exhibited saccadic and pursuit abnormality, respectively, while none of the control subjects had either abnormality. A recent cross-sectional study of pediatric patients (aged 11 to 17 years) found that 29% of these patients had saccadic dysfunction [28]. A recent meta-analysis showed that memory-guided and anti-saccades were more prominent saccadic dysfunction in mTBI patients. These saccades require a higher cognitive load as they utilize executive control from different brain areas [29]. Associated saccadic and pursuit dysfunction symptoms include poor reading and tracking ability, which impact learning and reading efficiency. Saccadic eye movement generation involves multiple brain centers. Visuospatial information is relayed from the occipital lobe and executed in the parietal and frontal lobes saccadic center, including the frontal eye field, the dorsolateral prefrontal cortex, the supplementary motor area, the cingulate eye field, and the parietal eye fields [30]. Thus, the nature of DAI in mTBI renders these brain areas susceptible to damage, causing versional eye movement dysfunction.

The most frequent vergence anomaly in mTBI is convergence insufficiency (CI). The prevalence of CI in the general population is estimated to be approximately 7.7% to 8.3% [31,32]. However, the prevalence of CI after mTBI has been shown to be much higher. Capo’-Aponte reported that 25% of the non-blast mTBI group had CI. A recent study of the prevalence of concussion-related vision problems in children and adolescents found that 49% of this population had CI [28]. Using objective measurements, Scheiman et al. found that the convergence eye movement was markedly impaired in a group of concussed individuals. Dynamic assessment of vergence eye movement showed peak velocity and accuracy abnormalities [33]. Common associated CI symptoms are headache, eye strain, diplopia, and loss of place while reading [22,34]. Other less common vergence dysfunctions include convergence excess, divergence excess, and divergence insufficiency [34]. Because the vergence system is coupled with accommodation during near response, vergence issues are often accompanied by accommodative abnormalities.

## 5. Accommodative System Control

### 5.1. Static Accommodation

**Figure 2:**
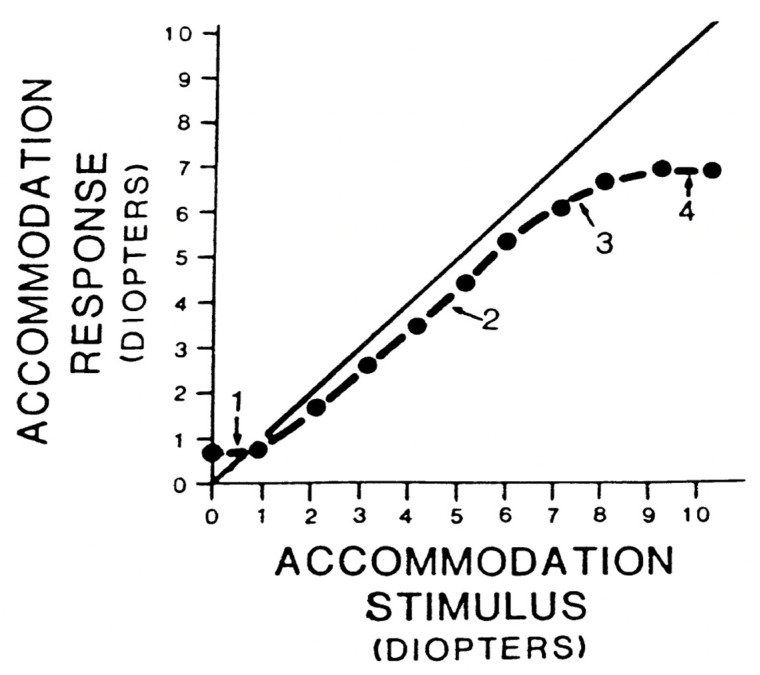
The stimulus–response function. Reproduced with permission from Hung, G.K., Ciuffreda, K.J., Khosroyani, M. et al., *Models of Accommodation*, Springer Nature, 2002 [36].

The basic accommodation static behavior can be quantified by plotting the accommodative response (AR) versus the accommodative stimulus (AS), as shown in Figure 2. In the ideal optical system, AR is equal to AS. However, the accommodation can exhibit a lead or lag of accommodation, resulting in an accommodative error (AE). Therefore, the AR/AS relationship does not fall on the 1:1 line. As can be seen in Figure 2, the stimulus–response curve can be divided into four zones: (1) the initial nonlinear portion of the curve from 0 to 1.5 D of AS, (2) the linear zone over which a change in AS produces a proportional change in AR, (3) the nonlinear transition zone (region of soft saturation), and (4) a nonlinear zone (hard saturation) that defines the amplitude of the accommodation [35].

**Figure 3:**
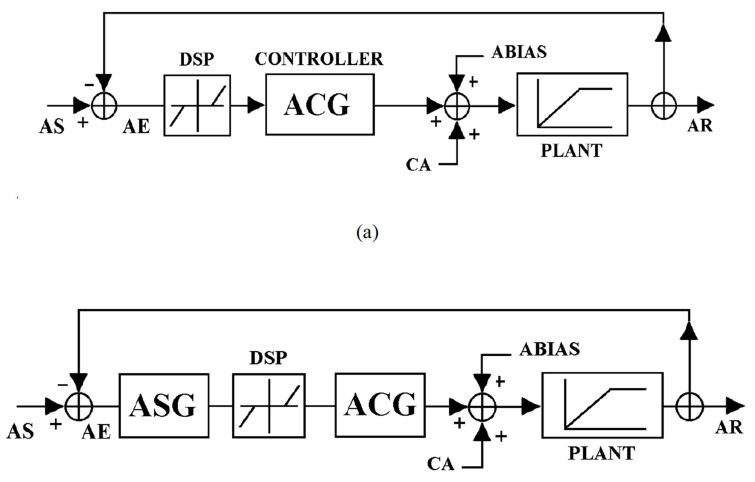
(**a**) Hung and Semlow simplified accommodation model. (**b**) A diagram of the modified control theory model of steady-state accommodation. The accommodative sensory gain (ASG) represents the error signal’s degradation in the sensory part of the system placed before the dead space (DSP), a nonlinear operator representing the oculomotor controller’s threshold [36]. Reproduced with permission from Hung, G.K., Ciuffreda, K.J., Khosroyani, M. et al., *Models of Accommodation*, Springer Nature, 2002 [36].

Due to the complexity of the accommodation neural network, accommodation bioengineering models have been developed to represent the static behavior of the accommodation response. These models provided a logical framework for understanding the behavior of accommodation. A simplified version of Hung and Semmlow’s classic static accommodation model is shown in Figure 3a. In Figure 3a, the difference between AR and AS results in an AE or the retinal image defocus signal. This error signal is input to dead-space operator (DSP), also known as the depth of focus. If AE exceeds the limit of DSP, the error signal could proceed to drive the accommodative controller (ACG). The ACG output is summed with the tonic accommodation (ABIAS) and vergence accommodation (assuming an open-loop vergence) to drive the plant, e.g., ciliary muscle, zonules and crystalline lens, eliciting the accommodative response [37]. The error signal is fed back to the accommodation system through a negative feedback loop to maintain a steady state of accommodation in the presence of a visual stimulus.

Hung and Semlow’s model attributed the signal degradation to the ACG element. However, other accommodative stimulus conditions, such as luminance [38], spatial frequency, and contrast, have also been shown to affect the accommodation response [39,40]. To account for the sensory component of the accommodative system control induced by changes in stimulus conditions, Jiang (1997) proposed a modification to the classic steady-state accommodation model [37,41]. Accommodative sensory gain (ASG) was introduced along with the DSP in the feed-forward accommodation loop. A diagram of the modified model is illustrated in Figure 3b. The combination of DSP and ASG represents the effective threshold to blur signal (ET) of the optical system and the accommodative neuronal system. This ET would represent the sensitivity to the blur signal needed to drive the accommodative output [41,42].

### 5.2. Dynamic Accommodation

Dual-mode control model of accommodation was developed to explain the dynamic accommodation behavior. The underlying principles of this model were based on experimental evidence that the vergence system behaves differently in response to step and ramp stimuli. The accommodation system showed that it behaves similar to the vergence system, indicating the potential presence of dual-mode characteristics. The accommodation response to ramp stimuli below 1.5 D/sec in their experiment showed a smooth tracking movement. In contrast, the response of ramp stimuli above this threshold exhibited a staircase-like step behavior, similar to that for the vergence system [43].

**Figure 4:**
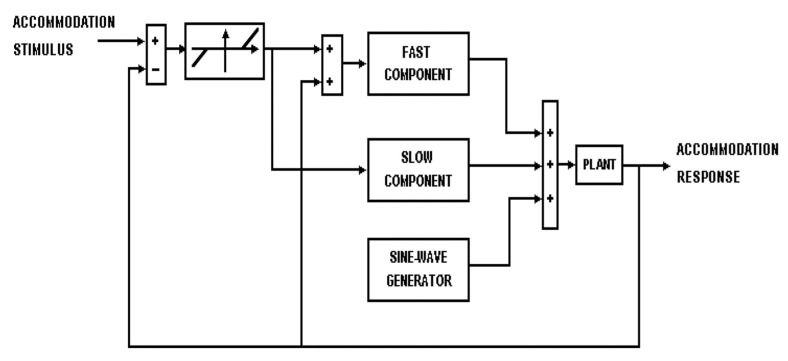
A diagram of the dual-mode accommodation model. Reproduced with permission from Hung, G.K., Ciuffreda, K.J., Khosroyani, M. et al., *Models of Accommodation*, Springer Nature, 2002 [36].

The dynamic accommodation operates under two modes (Figure 4). The first is fast open-loop mode, which accounts for the relatively rapid increase in step response. The second is a slow component that provides closed-loop feedback and accounts for accurate and stable steady-state accommodation. The dual-mode control model also uses a sensory property, a sampler, and predictor elements. The sampler operates under conditions when the accommodation velocity changes above a certain threshold. The predictor is a calculating unit that uses velocity and position to estimate the target’s future position and produce the calculated accommodative response [44].

## 6. MTBI Impact on the Accommodative System

Similar to vergence dysfunctions, accommodative anomalies in mTBI occur at a rate significantly higher than in the general population. The most commonly reported deficits include accommodative insufficiency and accommodative infacility, with accommodative spasm and accommodative excess reported less frequently. Epidemiological reports estimate that approximately 35% to 51% of individuals with mTBI experience accommodative insufficiency [22,28], reflecting the system’s vulnerability to diffuse neural injury. Notably, many early studies relied on subjective clinical metrics, which limited the precision of diagnosis and understanding of the underlying neural mechanisms.

Only a few studies have employed objective, instrument-based assessments to characterize the nature and extent of accommodative dysfunction following mTBI. Green et al. [45] and Thiagarajan and Ciuffreda [46] were among the first to report deficits in static and dynamic parameters, including increased accommodative lag, reduced peak velocity, and prolonged response latency. These findings strongly suggested that mTBI may compromise both the sensory input mechanisms (e.g., blur signal detection) and motor output systems (e.g., ciliary muscle control).

The body of evidence has continued to grow with additional studies employing larger and more diverse samples. Dutta et al. found that individuals with mTBI demonstrated significantly reduced accommodative responses, along with slower pupillary constriction velocities—a dual deficit pointing toward dysfunction at both the cortical and subcortical levels, possibly involving parasympathetic disruption [47,48]. In the pediatric population, Wiecek et al. identified reduced accommodative amplitude in more than half of concussed participants [49]. Importantly, these deficits frequently co-occurred with convergence and tracking impairments, highlighting the interconnected nature of oculomotor control systems in mTBI.

Dynamic aspects of accommodation have received increasing attention due to their relevance in real-world visual tasks. Haensel et al. used a low-frequency sinusoidal stimulus to probe dynamic accommodation and found that children with concussion exhibited a reduced amplitude of accommodative response, especially in the absence of disparity cues. These findings underscore a disruption in accommodative tracking mechanisms, possibly reflecting impairments in both feedforward prediction and feedback correction loops [50].

Beyond response amplitude and latency, emerging research has begun to investigate the stability and precision of the accommodative system. Almutairi et al. analyzed accommodative microfluctuations—a marker of steady-state motor control—and found significantly diminished low- and high-frequency components (LFCs, HFCs) in individuals with mTBI. These changes were especially prominent at higher stimulus levels and were negatively correlated with accommodative error (AE), suggesting impaired fine motor modulation and reduced dynamic flexibility of the lens system [51].

A comprehensive review by Thiagarajan and Ciuffreda consolidated these findings and concluded that mTBI is associated with widespread alterations across both static and dynamic accommodation domains. Importantly, these deficits appear to be responsive to structured vision therapy, supporting the role of neural plasticity and rehabilitation in visual recovery. Their findings serve as a foundation for applying model-based therapy approaches in this population [52].

Accommodative dysfunctions in mTBI are not only measurable but also functionally significant. Affected individuals frequently report headache, blurred near vision, and visual fatigue, particularly during sustained near work. These symptoms can substantially impair quality of life and interfere with academic, occupational, and daily tasks requiring prolonged visual concentration.

A summary of recent studies investigating objective accommodative dysfunction in mTBI, along with sample characteristics, test parameters, and key findings, is provided in Table 1 below.

### 6.1. Accommodation Model and mTBI

The accommodation model has helped us further understand the accommodation’s basic mechanisms and its application to clinical conditions such as accommodative dysfunction, amblyopia, and myopia. These abnormalities can be due to the accommodation’s sensory and/or motor systems. For example, Ciuffreda found that the reduced accommodative function in amblyopia reflects the sensory loss and the reduction in overall sensitivity to blur [53,54]. Accommodation sensory impairment was also found in individuals with late-onset myopia. Using the modified accommodation control, Jiang found that the ET was significantly elevated in subjects with late-onset myopia compared to emmetropes [41]. In addition, reduced blur sensitivity to retinal defocus has been found in individuals with myopia [55].

The specific contribution of the accommodation’s sensory and motor systems to the accommodative deficits in mTBI has not been studied. Given that diffuse axonal injury (DAI) in mTBI often disrupts white matter pathways involved in visual processing, it is plausible that both sensory and motor components of the accommodation control system are affected. Damage to regions involved in blur detection (e.g., visual cortex, parietal lobes) may elevate the effective threshold (ET), while impairments in frontal–striatal pathways could slow the motor response, manifesting as reduced peak velocity and increased latency. These disruptions align closely with the ASG and ACG components described in accommodation control models. The contribution of abnormalities in the dynamics of fast and slow components of the accommodation control system to these observed dynamic accommodation deficits is still unclear.

### 6.2. Clinical Implications: Using Accommodation Models to Guide Vision Therapy in mTBI

Optometric vision therapy for accommodative dysfunctions has a long-standing scientific foundation and demonstrates high success rates in both children and adults [56]. Drawing upon bioengineering models of the accommodative system—specifically static and dynamic control frameworks—therapy regimens can be precisely designed to rehabilitate sensory and motor deficits commonly seen in mild traumatic brain injury (mTBI). These models provide a thorough conceptual framework that links diverse clinical presentations to underlying neurological or biomechanical deficits, enabling targeted therapies.

The static accommodation model highlights two critical components related to mTBI the depth of focus (DSP) and accommodative sensory gain (ASG), which reflect the sensory pathway’s responsiveness to blur, as well as accommodative controller gain (ACG), which governs motor output to the ciliary muscle–lens complex. Patients with mTBI often have heightened accommodation lag, signifying less sensory responsiveness to blur cues or compromised motor gain. Therapeutic methods such as blur discrimination training, sequential lens sorting, and low-contrast letter acuity tasks are employed to recalibrate (DSP) and (ASG), while monocular/binocular flipper therapy (utilizing ±1.50 D or ±2.00 D lenses) seeks to improve the ACG by fostering precise and consistent accommodation responses.

In parallel, the dynamic accommodation model, particularly the dual-mode control model, sheds light on the velocity and timing deficits in mTBI patients. Studies have shown that individuals with mTBI exhibit reduced peak velocities, increased time constants, and prolonged latency, reflecting dysfunction in both the fast (open-loop) and slow (closed-loop) accommodative control systems. Therapeutic techniques such as step blur (jump focus) stimuli and ramp blur exercises can activate both components [57]. The fast system, linked to anticipatory, feedforward control, encounters abrupt blur change, whereas the slow system, tasked with ensuring steady fixation, is activated through prolonged near-point activities that facilitate error correction through feedback processes.

These therapeutic methods have been demonstrated to elicit actual physiological alterations, encompassing enhancements in time constant, latency, and controller gain, as objectively assessed by autorefractors and dynamic optometers. These enhancements indicate fundamental oculomotor plasticity, which is of significant importance in neuro-rehabilitation after mild traumatic brain injury (mTBI) [57,58,59]. Furthermore, vision therapy incorporates multimodal cueing (such as kinesthetic and auditory biofeedback), attentional modulation, and behavioral relaxation to improve motor learning and generalize accommodative control to practical visual requirements.

Neuroimaging studies utilizing functional MRI support these physiological findings, demonstrating that accommodative and vergence therapy can induce quantifiable alterations in oculomotor brain areas. Enhanced functional activity and coordination have been noted in the frontal eye fields and oculomotor vermis, alongside improved disparity processing in the posterior occipital brain. These neural changes substantiate the evidence for therapy-induced plasticity and emphasize the potential of targeted interventions to facilitate cortical reconstruction after mTBI [60,61,62].

In summary, the application of accommodation models enables clinicians to diagnose subcomponent dysfunctions in mTBI-related accommodative anomalies and design component-specific therapy protocols. Readers are encouraged to refer to this review for an in-depth analysis of how static and dynamic accommodation models inform clinical strategies for vision therapy in mTBI [63].

### 6.3. Factors Influencing Variability in Visual System Recovery After mTBI

Accommodative dysfunction frequently occurs after mild traumatic brain injury (mTBI), yet recovery trajectories and symptom severity exhibit significant variability among individuals. Age, injury severity, pre-existing visual or neurological conditions, and the type of rehabilitation interventions contribute to this variability. Research indicates that older adults and individuals with prior concussions or chronic health conditions may experience prolonged or complex recovery patterns due to reduced neuroplasticity or compounding deficits [64]. Injury severity plays a crucial role; visual field deficits and complex oculomotor abnormalities are more frequently associated with moderate to severe TBI compared to mild cases, underscoring the impact of the extent of neural disruption on visual outcomes [65]. Additionally, pre-existing health conditions, especially those related to the central nervous system, may interact with TBI-related impairments in complex ways, influencing recovery potential [66]. Finally, a recent systematic review highlights the significance of personalized rehabilitative approaches; responses to vision therapy and oculomotor re-training differ based on individual factors, such as initial visual status, cognitive load tolerance, and adherence to therapy [67]. These findings highlight the necessity for a personalized approach to assessment and intervention in the management of accommodative dysfunction in mTBI populations.

## 7. Limitations

Although this study offers a thorough synthesis of present data on accommodative dysfunction in mTBI, some shortcomings should be noted. First, the generalizability of results is constrained by small sample sizes and varied approaches used in much of the extant research. Still, rather few are objective evaluations of accommodation; differences in diagnostic criteria and testing methods among research impede direct result comparison. Furthermore, although this study underlines the possible significance of both sensory and motor disturbances depending on bioengineering models, the relative contributions of these components have not been scientifically examined in relation to mTBI.

In addition, our knowledge of dynamic accommodation deficits is always changing and there is no longitudinal evidence on recovery paths. This study mostly addresses adult populations; so, its relevance to pediatric or geriatric groups may be restricted. Though theoretical models of accommodation are thoroughly examined to connect knowledge with clinical findings, empirical validation of these models in mTBI populations is still scarce.

Future research should fill these holes by means of well-controlled, large-scale trials following standardized procedures and should investigate the longitudinal development of accommodative dysfunction, including its sensitivity to focused vision treatment.

## 8. Conclusions

Mild traumatic brain injury significantly impacts the visual system, with oculomotor dysfunctions, especially in accommodation, being a common and debilitating consequence. The accommodative system’s dependence on accurately calibrated sensory and motor functions makes it especially susceptible to diffuse axonal injury in mild traumatic brain injury (mTBI). Despite progress in characterizing accommodative deficits, the distinct roles of sensory and motor dysfunctions in contributing to accommodative lag and abnormalities in dynamic responses remain unclear.

## Figures and Tables

**Table 1:** Summary of studies investigating accommodative dysfunction in mild traumatic brain injury (mTBI). Abbreviations: AA = accommodative amplitude; PRA/NRA = positive/negative relative accommodation; AE = accommodative error; LFC = low-frequency component; HFC = high-frequency component; TA = tonic accommodation.

Author (Year)	Sample Size (mTBI/Control)	Age Range/Mean	Type of TBI	Duration Since Injury	Accommodation Type (Static/Dynamic)	Parameters Tested	Key Findings
**Green et al. (2010)** [45]	12/10	18–40 years	mTBI	Varied (not specified)	Static and Dynamic	AA, accommodative interactions, peak velocity, fatigue	Reduced AA, abnormal interactions, slowed dynamics with fatigue; both static and dynamic deficits noted
**Thiagarajan and Ciuffreda (2014)** [46]	12/10	23–33 years (mean 29)	mTBI	>1 year	Static and Dynamic	Amplitude of Accommodation(AA), PRA/NRA, peak velocity, time constant	Reduced AA (~1.5D), reduced PRA/NRA, decreased peak velocity, prolonged response time; improved post-training
**Chen et al. (2020)** [48]	22/22	18–38 years (mean ≈ 27.2)	TBI (Mild and Severe)	0.17–84 months	Static	Accommodative amplitude, accommodative facility, accommodative lag (2–5 D), variability, stereopsis	Significantly reduced AA and facility; greater accommodative lag and variability across stimulus levels; 32% showed accommodative insufficiency; strong correlation with symptom severity
**Wiecek et al. (2021)** [49]	116/—	5–21 years (median 15)	Concussion (mTBI)	≥21 days	Static	Accommodative amplitude, near point of convergence	63/116 showed reduced accommodative amplitude; correlated with convergence and tracking deficits
**Dutta et al. (2024)** [47]	63/90	18–35 years	mTBI	≥6 months	Static (response magnitude)	Accommodative response (diopters), pupillary dynamics	Reduced accommodative response (~−1.12 vs. −1.39 D); also reduced pupillary constriction velocities
**Haensel et al. (2024)** [50]	32/32	14.4 ± 2.6 (mTBI), 12.7 ± 2.1 (controls)	Concussion (mTBI)	36–273 days (mean 107)	Dynamic	Amplitude of accommodative response to moving target (0.1 Hz)	Reduced monocular accommodative response in mTBI; no vergence differences; disparity-driven response
**Almutairi et al. (2025)** [51]	30/54	18–33 (mTBI), 20–30 (controls)	mTBI	Up to 5 years	Static (Steady-State)	Accommodation Microfluctuations (LFC, HFC), Tonic Accommodation (TA)	Lower MFs (LFC and HFC) in mTBI, especially at high stimulus levels; strong AE–LFC negative correlation suggests impaired motor control

## Data Availability

Not applicable.
